# Effects of Konjac Glucomannan and Chitin Nanowhiskers on Structural and Physical Properties of Soy Protein Isolate Composite Hydrogels

**DOI:** 10.3390/foods14050767

**Published:** 2025-02-24

**Authors:** Jianbo Zhao, Danjie Li, Ronghua Deng, Jie Pang, Cailing Tong, Chunhua Wu

**Affiliations:** 1 College of Food Science, Fujian Agriculture and Forestry University, Fuzhou 350002, China; zhjb19970213@163.com (J.Z.); 15216948080@163.com (D.L.); pang3721941@163.com (J.P.); 2 College of Mechanical and Electrical Engineering, Wuyi University, Wuyishan 354300, China; 3 Fuzhou Sotia Food Co., Ltd., Fuzhou 350002, China; dengronghua66@163.com

**Keywords:** rheological characteristics, oxidized chitin nanocrystal, composite hydrogels, mechanical properties, protein–polysaccharide interaction

## Abstract

Soybean protein isolates (SPIs) have been widely studied because of their excellent gel-forming properties. However, their unstable gel structures and poor strength limit their applications in the food industry. To address this, konjac glucomannan (KGM) and oxidized chitin nanocrystals (O-ChNCs) were introduced into SPI-based hydrogels to enhance their mechanical properties. The present study investigated the effects of incorporating KGM and O-ChNCs on the physical properties and microstructure of SPI hydrogels, as well as the possible underlying mechanisms. The rheological behavior test of the solution demonstrated that the viscoelastic properties of the sol were enhanced upon incorporating O-ChNCs and KGM. Scanning electron microscopy showed highly compact and uniformly distributed SPI hydrogels with the addition of O-ChNCs and KGM. Gel strength and textural property tests showed that the gel strength and gel hardness of SPI hydrogels with the addition of O-ChNCs and KGM were 102.57 ± 1.91 g/cm^2^ and 545.29 ± 6.84 g. O-ChNCs effectively filled the SPI hydrogel network, while KGM enhanced physical entanglement between SPI molecular chains and formed intermolecular hydrogen bonds. Therefore, this study provides an important basis for the introduction of SPI-based hydrogels in the biomedical and food industries.

## 1. Introduction

Soy protein isolates (SPIs) are a key class of plant proteins widely used in food applications because of their rich nutritional value and functional properties [1]. A key function of SPIs is their ability to form gels. Through thermal induction, SPIs undergo molecular aggregation and unfolding, resulting in cross-linking to form a 3D gel network [2]. When the critical gel concentration is reached, SPIs can form gels via simple heat treatment. However, there are shortcomings, such as the necessity of a high protein concentration, weak gel strength, and instability of the gel structure, which limit its further application in the food industry [3].

In recent years, researchers have successfully introduced various polysaccharides, such as corn fiber gum, κ-carrageenan, and citrus pectin, into SPI matrices to form composite hydrogels, which enhance the structural stability of protein gels [2,4,5]. Konjac glucomannan (KGM) is a natural polysaccharide extracted from the tubers of the *Amorphophallus konjac* plant [6]. It is composed of polymerized β-(1–4) glycosidic linkages of D-glucose and D-mannose in a molar ratio of 1:1.6 or 1:1.4 [7]. KGM possesses excellent gelation, favorable film-forming abilities, high thickening properties, satisfactory biocompatibility and biodegradability, and non-toxicity. KGM is considered a safe food additive by the U.S. Food and Drug Administration [8]. KGM can form terminally intractable gels by removing some of its acetyl groups using alkali or basic salts and heat. The stability of composite hydrogels has shown synergistic interactions when pea isolate, soy, and myofibrillar proteins are incorporated into KGM gels [9,10,11]. In KGM and SPI composite hydrogels, intermolecular hydrogen bonding enhances the network structure and gel hardness [12]. However, the characteristic physical properties (gel strength and water-holding capacity) of SPI and KGM composite hydrogels are still somewhat deficient.

Nanofillers are potential materials that can be used to address this challenge owing to their nanoscale size and excellent filling properties. Oxidized chitin nanocrystals (O-ChNCs) have been synthesized through the process of the TEMPO-mediated oxidation of chitin [13]. O-ChNCs retain the original properties of chitin while also exhibiting excellent properties. O-ChNCs have a great influence on the structural composition and functional characteristics of hydrogels [14]. When added to a KGM-based matrix, O-ChNCs are well dispersed and form intermolecular hydrogen bonds, enhancing the mechanical performance of the resulting hydrogels [6]. However, there are no reports on the effects of KGM and O-ChNCs on the mechanical properties of SPI hydrogels. SPIs and KGM form intermolecular hydrogen bonds and electrostatic interactions, while O-ChNCs, with their numerous hydroxyl groups and high surface charge, form physical interactions (hydrogen bonding, electrostatic interactions, and hydrophobic interactions) with SPIs and KGM, thus improving the properties of the composite hydrogels.

The main objective of this study was to evaluate the effects of KGM and O-ChNCs on the physical and structural properties of SPI-based hydrogels. Based on the properties of the composite hydrogels, a mechanism was proposed to explain the formation and stability of SPI-based hydrogels enhanced with KGM and O-ChNCs. This study provides an important basis for the application of SPI-based hydrogels in the biomedical and food industries.

## 2. Materials and Methods

### 2.1. Materials

SPIs were provided by Linyi Shandong Biological Products Co., Ltd. (Linyi, China). Chitin (practical grade; degree of acetylation: 90%; derived shrimp shell) was obtained from Sigma-Aldrich Chemical Co. (St. Louis, MO, USA). KGM (purity ≥ 95%) was obtained from Shanghai Brilliant Gum Co., Ltd. (Shanghai, China). Na_2_CO_3_, urea, NaCl, and sodium dodecyl sulfate (SDS) were analytical-grade chemical reagents, provided by Sinopharm Group Chemical Reagent Co., Ltd. (Shanghai, China).

### 2.2. Preparation of Composite Hydrogels

O-ChNCs were prepared following a method which was previously delineated in our extant study [13]. The preparation of hydrogels was based on previous studies by He et al. [15] and Li et al. [6] with minor modifications. A certain amount of O-ChNC powder was dispersed in deionized water and ultrasonicated at 50 W for 10 min. A certain amount of SPI powder was dissolved in deionized water and mechanically stirred (500 rpm) for 90 min in a water bath at 40 °C. Subsequently, 10 mL of a 4.5% Na_2_CO_3_ solution (*w*/*v*) and a specified amount of KGM were added to the SPI solution and stirred (650 rpm) for 60 s. Finally, the sample volume was adjusted to 100 mL. The samples were injected into cylindrical silica gel molds (30 mm × 20 mm in height) and heated at 90 °C for 90 min, then cooled. Pure SPI hydrogels were named Con, and the obtained composite gels were denoted as SOC, SK, and SKOC, corresponding to the addition of O-ChNCs, the addition of KGM, and the addition of O-ChNCs and KGM in SPI hydrogels. The compositions of the composite hydrogel samples are listed in Table 1.

### 2.3. Rheological Behavior

The rheological behaviors of the composite hydrogel samples were tested using a rotational rheometer (MCR301, TA, New Castle, DE, USA), following the method of Li et al. [6], with minor modifications. The rheometer was equipped with PP50 plate probes, set at a plate-to-plate distance of 1.0 mm. The excess sample was wiped off the edges of the plate and left in place for 1 min. All samples were subjected to three parallel experiments.

#### 2.3.1. Steady Shear Properties

Samples were tested at room temperature (25 ± 1 °C) with a shear rate of 0.1–100 s^−1^. The rheological behavior was modeled using the cross-model Equation (1):

(1) $$\eta =\frac{\eta_{0}-\eta_{\infty }}{{\left[1+\left(\lambda_{C}\gamma \right)\right]}^{n}}+\eta_{\infty }$$

where *η* represents the viscosity (Pa·s), *η*_0_ represents the zero-shear rate viscosity (Pa·s), *η_∞_* denotes the infinite shear rate viscosity (Pa·s), *λ_c_* is a time constant (s), *γ* represents the shear rate (1/s), and *n* is a dimensionless constant.

#### 2.3.2. Dynamic Oscillatory Characterization

Initially, the linear viscoelastic region of the samples was ascertained through small amplitude oscillatory strain (SAOS) sweep tests at a temperature of 25 °C. These tests were conducted using strain values ranging from 0.01% to 100%, at a frequency of 1 Hz. Subsequently, a strain amplitude of 1%, within the linear viscoelastic domain, was selected for all subsequent SAOS tests. A frequency sweep test was then conducted, encompassing frequency values between 0.1 and 100 rad/s. Dynamic ramping scans were performed at a frequency of 1 Hz for 2 °C/min from 25 to 90 °C.

### 2.4. Color Measurement

The color of the samples was detected using a Precision Colorimeter (CS-200; Hangzhou, China). The whiteness was calculated using the following Equation (2):

(2) $$\mathrm{Whiteness}=100-\sqrt{{\left(100-L*\right)}^{2}+{a*}^{2}+{b*}^{2}}$$

where L* represents luminance, a* represents red/green, and b* represents yellow/blue.

### 2.5. Scanning Electron Microscopy (SEM)

The microstructures of the cross-sections of the Con, SOC, SK, and SKOC samples were obtained using SEM (Japan Electron Optics Laboratory Co., Ltd., Tokyo, Japan). The Con, SOC, SK, and SKOC hydrogels were lyophilized in a vacuum freeze dryer (FDL-2000, EYELA Co., Ltd., Tokyo, Japan). Subsequently, the samples of SPIs, SOC, SK, and SKOC from the lyophilized gels were broken in liquid nitrogen and sprayed with gold. The fracture surface was examined using SEM at a magnification of 100×, with an accelerating voltage of 5.0 kV. Meanwhile, the average pore size of the hydrogel was analyzed by measuring all the pores in the corresponding SEM images using the Nano Measure 1.2.5, and the measurement of the pore size was repeated three times [16].

### 2.6. Fourier Transform Infrared (FT-IR) Spectroscopy of Composite Hydrogels

An FT-IR spectroscope (Bruker VERTEX 70, Waltham, MO, USA) was used to characterize the molecular structures of the lyophilized hydrogels. Approximately 5 mg of the lyophilized Con, SOC, SK, and SKOC hydrogels was weighed, pulverized, and mixed with KBr powder at a 1:100 (*w*/*w*) ratio for grinding and tableting. The samples were scanned in the range of 4000 to 400 cm^−1^.

The spectra of the samples were processed using OMNIC 9.0 and Peak Fit v4.12. Baseline shifts and deconvolution were performed using the Fourier method, followed by second-order derivative calculations and then fitting to a Gaussian curve [17]. The secondary structure composition of each protein was ascertained by dividing the peak area of the protein structural constituent by the total peak area of the amide I region (1600–1700 cm^−1^).

### 2.7. X-Ray Diffraction (XRD)

The crystal structures of the Con, SOC, SK, and SKOC lyophilized hydrogels were analyzed using X-ray diffractometry (XRD, Brucker D8 Advance, Berlin, Germany). Cu Kα radiation, with a wavelength of 1.54056 Å, was employed. Spectral intensities were measured over a range of 5° to 70° at a scan speed of 2°/min as a function of 2θ.

### 2.8. Thermal Gravimetric Analysis (TGA)

A TGA (STA449C/6/G*; Netzsch, Selb, Germany) of the Con, SOC, SK, and SKOC lyophilized samples was performed using the method described by Tong et al. [18]. The samples were scanned from 30 °C to 600 °C at a rate of 10 °C/min in a nitrogen atmosphere.

### 2.9. Water Holding Capacity (WHC)

The WHC of the composite hydrogels was determined by the method of Gao et al. [19] with slight modifications. A certain quantity of sample was loaded into a centrifuge tube and centrifuged at 10,000 rpm for 15 min at 4 °C (H2-16KR, Suzhou, China). The supernatant was then poured off. The WHC of the hydrogels was calculated using Equation (3).

(3) $$\mathrm{WHC}(\%)=\frac{W_{2}-W_{0}}{W_{1}-W_{0}}\times 100\%$$

where *W*_2_ represents the total mass after centrifugation (g), *W*_1_ represents the total mass before centrifugation (g), and *W*_0_ indicates the mass of the centrifuge tube (g).

### 2.10. Textural Properties and Gel Strength

A texture analyzer (TA-XT plus; Stable Micro Systems, Godalming, UK) was used to determine the gel strength of the samples. A P/5 probe was used to analyze the compressive strength of the samples. The compression mode was set with a compression depth of 10 mm (1 mm/s), and the trigger force was 5.0 g. In addition, textural properties were assessed using a P/36 probe. The compression rate was set to 1 mm/s, the trigger force was 5.0 g, and the compression depth was fixed at 80%. The recorded data included the hardness, cohesiveness, springiness, and chewiness.

### 2.11. Measurement for Molecular Interaction Forces

We evaluated the molecular interactions using the methodology outlined by Odelli et al. [9], with certain adaptations. The Con, SOC, SK, and SKOC samples were immersed in 100 mL distilled water (control group), 100 mM NaCl, 100 mM urea, and 100 mM SDS solutions and kept at 37 °C and 100 rpm for 24 h. The hydrogels were removed, and the surface water was wiped off. Subsequently, the gel strength of the hydrogel was assessed using a texture analyzer.

### 2.12. Statistical Analysis

All tests were executed in three replicates. The results are shown as the mean ± standard deviation (SD). Statistical analyses were performed using Origin 2018 and IBM SSPS 26.0. An analysis of variance (ANOVA) and Duncan’s Multiple Range Test were used to compare the differences among all hydrogels, with statistically significant differences set at *p* < 0.05.

## 3. Results and Discussion

### 3.1. Color and Appearance of Hydrogels

**Figure 1:**
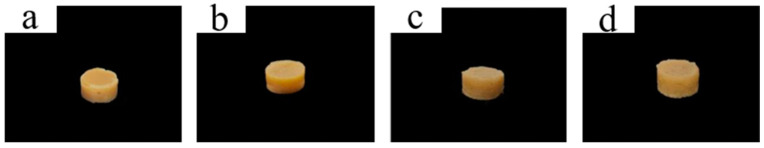
The appearance of Con (**a**), SOC (**b**), SK (**c**), and SKOC (**d**) hydrogels.

The appearance of the hydrogels is shown in Figure 1. All hydrogels were in a gel-like solid state with a smooth surface and no slumping. All four hydrogels were yellowish-brown in color. This may be due to the yellow color of the protein itself, as well as the partial denaturation of the protein by Na_2_CO_3_, which causes the oxidation of -NH_2_ groups, making them turn them yellow. Table 2 lists the color parameters of the hydrogels. Higher L*, a*, and b* values indicate higher brightness, redness, and yellow color values, respectively. The Con and SOC hydrogels had higher brightness and yellow color values. Previous studies have shown that SPIs can increase the brightness and yellow values of the system [20]. The incorporation of KGM into SPI hydrogels resulted in a substantial decline in both L* (26.99 ± 0.18) and whiteness (26.54 ± 0.14). This was probably due to the deacetylation of KGM in an alkaline environment [21]. When O-ChNCs were added to the composite hydrogels, the b* value of the composite hydrogels decreased, possibly because the O-ChNCs were translucent and suspended in water [1].

### 3.2. Rheological Properties of Hydrogels

**Figure 2:**
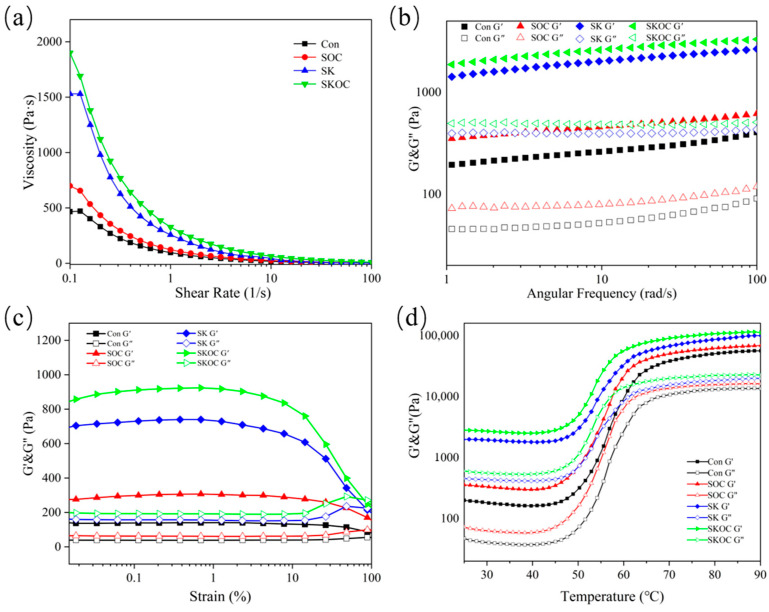
Rheological properties of Con, SOC, SK, and SKOC sols. Steady shear (**a**), angular frequency sweep (**b**), strain sweep (**c**), and temperature sweep (**d**).

The relationship between the shear rate and the apparent viscosity of the hydrogels is illustrated in Figure 2a. The apparent viscosity of the SPI-based hydrogels decreased with an increasing shear rate within the range of 0.1–100 s^−1^. This phenomenon was consistent with the behavior exhibited by pseudoplastic non-Newtonian fluids [22]. An increase in the shear rate results in the disruption of polymer molecular chains, leading to the reorganization of the chain orientation in the flow direction [23]. The apparent viscosities of the composite sols were greater than those of the SPI sols, with the SKOC sols having the highest apparent viscosity. The favorable thickening properties of KGM and the elevated modulus of elasticity of O-ChNCs led to the formation of a robust network structure between the molecular chains of SPIs, KGM, and O-ChNCs in the solution [24]. This result is consistent with the SEM results, where SKOC has a tighter network structure with smaller gaps. This process decreased the mobility of the molecular chains and increased the viscosity of the system [18,25]. 

The storage modulus (G′) indicates the elasticity and solid-like nature of the polymer, while the loss modulus (G″) indicates the viscous and liquid-like nature of the polymer. Figure 2b demonstrates the relationship between the amplitudes and viscosities of the samples. The G′ and G″ of hydrogels (G′ < G″) did not change with increasing shear strain over a certain range of shear strain. This finding suggests that the system’s elasticity predominates at this juncture, manifesting “solid-like” characteristics. It is conceivable that the structural integrity of the gel system remains uncompromised within a specific range of shear strain. It was noteworthy that in the linear viscoelastic region, the G′ and G″ of SOC, SK, and SKOC sols were higher than those of SPIs. This shows that the O-ChNCs and KGM can effectively improve the structural strength of SPI gels by filling the gaps in the SPI hydrogels [26]. When the amplitude was increased further, G′ started to decrease. This decrease indicates that the sol reached its maximum deformation capacity and was unable to withstand larger amplitudes.

The correlation between angular frequency and viscoelasticity is shown in Figure 2c. G′ was consistently larger than G″ for several sols in the 1–100 rad/s range. The high solid content of the system resulted in a robust network structure for cross-linking between macromolecules, thereby ensuring exceptional elastic solid properties. It was observed that the G′ and G″ of the sols increased with frequency over time, indicating that they were both weak physical gels based on non-covalent interactions [27].

Figure 2d shows the relationship between the temperature and viscoelasticity. During the process of warming, SPI-based sols demonstrated a greater G′ than G″. This finding implied that they demonstrated enhanced resilience. There was a slight decrease in G′ with increasing temperature (25–40 °C), which can be attributed to the disruption of hydrogen bonds between molecules and between molecules and water within the system, resulting in a reduction in viscosity. When SPI-based sols were further heated from 40 to 65 °C, the shear modulus increased steeply, which can be attributed to the gelation of KGM and the gelation of SPIs [28]. The system approached a steady state (65–90 °C), attributable to increased temperature, which resulted in alterations in the spatial structure of the protein and an augmentation in the degree of cross-linking of the protein gel [29]. The G′ and G″ of the composite sols were greater than those of the Con sols, consistent with the observations reported by Yang et al. [27].

### 3.3. Microstructure of Hydrogels

**Figure 3:**
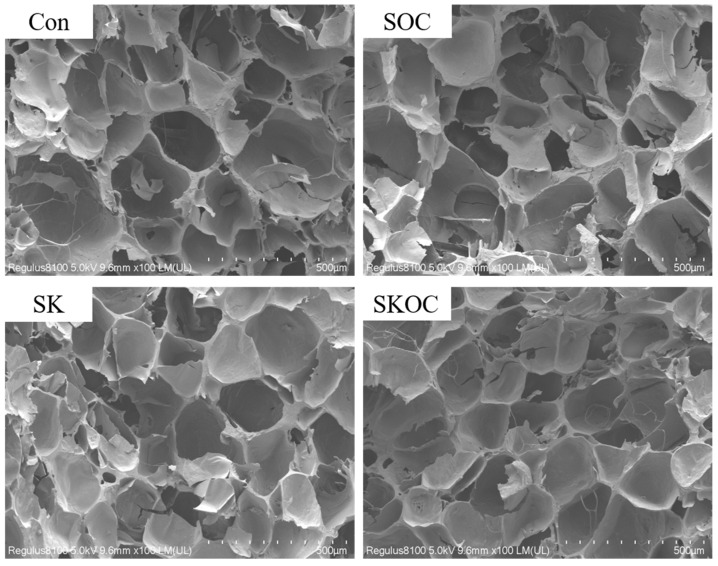
The microstructures of Con, SOC, SK, and SKOC hydrogels.

The effects of O-ChNCs and KGM on the microstructure of the SPI-based hydrogels were observed using SEM (Figure 3). The microstructures demonstrated that all the samples exhibited a honeycomb-like network structure, which imparted distinctive elastic properties to the hydrogels. During heating, protein chains unfold and aggregate to form a 3D network. The pores of the Con hydrogels were irregular, varied in size, and distinctly large (300.09 ± 49.48 μm), resulting in a loose and soft structure. The addition of KGM and O-ChNCs reduced the size of the hydrogel pores, and the pore size of the SKOC hydrogel was 184.94 ± 12.67 μm. He et al. [15] found that SPI-KGM composite hydrogels have poor aggregation and large pores, and the introduction of nanocellulose crystals can reduce the pore size of the gel network. The O-ChNCs effectively filled the SPI matrix and improved the network structure of the hydrogels, which concurred with the results of other studies [6]. KGM and SPIs are capable of forming a greater number of hydrogen bonds, which fill the voids within the SPI network and result in a more compact structure. Furthermore, the phenomenon of reduced pore size may also be attributed to the influence of long-chain polysaccharides, which influences the aggregation and bonding of expanded proteins, thereby bonding the entire protein and enhancing the composite hydrogel frameworks [11,30].

### 3.4. FT-IR Analysis of Hydrogels

**Figure 4:**
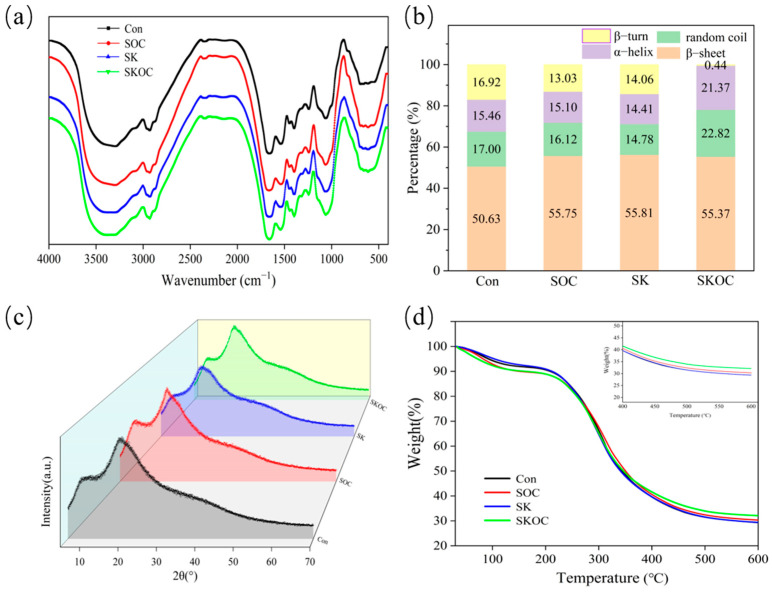
FT-IR spectra (**a**), secondary structure parameter (**b**), XRD patterns (**c**), and TGA profile (**d**) of Con, SOC, SK, and SKOC hydrogels.

FT-IR spectroscopy is commonly used to analyze the chemical structures of composite hydrogels. Figure 4a shows the FTIR spectra of the Con, SOC, SK, and SKOC hydrogels. The peaks in the 3700–3200 cm^−1^ range correspond to the bending vibrations of –OH stretching [31,32]. When KGM and O-ChNCs were added to the composite hydrogels, they weakened the broadband at 3700–3200 cm^−1^ and gradually shifted to higher wavenumbers. This phenomenon can be interpreted as resulting from the formation of hydrogen bonds [33]. The peaks at 1700–1600 cm^−1^, 1550–1450 cm^−1^, and 1430–1240 cm^−1^ correspond to amide I, amide II, and amide III, respectively [34,35,36,37]. The peaks of the amide groups of SPIs were also red-shifted, probably because of the interaction between the amino or amide groups of the protein molecules and the hydroxyl groups of KGM and O-ChNCs. These interactions changed the stretching vibrations of the amide groups [6]. 

Figure 4b shows the secondary structure of the SPI-based hydrogels according to peak fitting analysis. The back convolution of amide I (1600–1700 cm^−1^) can supply critical information regarding the secondary structure of proteins. The fitting of the amide I band showed that the SPI was enriched in a β-sheet structure, which is in agreement with Zhang [38]. Compared to the Con hydrogels, the composite hydrogels demonstrated an increase in the percentage of β-sheets and a decrease in the percentage of β-turns. This may be because the presence of polysaccharides (O-ChNCs and KGM) resulted in the exposure of a greater quantity of hydroxyl groups and the formation of a greater number of hydrogen bonds. This prompts the formation of highly entangled networks around the SPI, which then undergo physical cross-linking to form a mesh. This mesh impedes the transfer of heat through the proteins, thereby slowing down heat exchange during the heating process and allowing for the retention of more lamellar structures [39]. This conclusion is consistent with the findings of gel strength analysis. The SKOC hydrogel exhibited a greater proportion of α-helix and random coils in comparison to the Con hydrogels. The observed increase in α-helix content may be explained by the increased interactions between SPIs and KGM as well as O-ChNCs, which together contribute to the formation of a more compact gel structure.

### 3.5. XRD Analysis of Hydrogels

XRD is commonly used to characterize the crystal structure and biocompatibility of hydrogels. The XRD patterns of the SPI-based hydrogels are shown in Figure 4c. The SPI-based hydrogels exhibited weak peaks at approximately 11.4° and 19.3°. The addition of O-ChNCs resulted in sharper diffraction peaks for the hydrogels than those observed for the hydrogels without O-ChNCs. This phenomenon may be attributed to the enhanced crystal structure of the O-ChNCs, which enhanced the crystallinity of the SOC and SKOC hydrogels [40]. The intensity of the diffraction peaks of the SPI-based hydrogel decreased and broadened with the addition of KGM. This involved a change in the structural order of the SPI conformation, as well as the generation of more interactions and cross-links between KGM and SPIs [41]. It was also shown that KGM and O-ChNCs are compatible with SPIs in the system, thus enhancing the mechanical properties of the composite hydrogel.

### 3.6. TGA Analysis of Hydrogels

The thermal decomposition patterns of the SPI-based hydrogels are shown in the TGA plots in Figure 4d. The mass loss behavior of the SPI-based hydrogels was similar and can be divided into three stages. The first stage of weight loss occurred between approximately 30 and 150 °C, which may be due to the evaporation of water [42]. The second stage was characterized by significant weight loss at temperatures between 150 and 350 °C, which could be attributed to the breakage of the gel backbone and the thermal decomposition of the cross-linked network [43]. The third stage occurred between 350 and 600 °C, involving a further degradation of SPIs, KGM, and O-ChNCs to carbonization. Notably, the residual mass of the composite gels was higher than that of the SPI hydrogels, with the SKOC hydrogels having the highest mass. This phenomenon could be attributed to the incorporation of O-ChNCs and KGM, which increased hydrogen bonding interactions and influenced protein aggregation before gelation, thereby enhancing the stability of the gel structure [44].

### 3.7. Textural Properties and Gel Strength Analysis of Hydrogels

Mechanical properties are key attributes used in the food processing industry to evaluate and accept a product. Table 3 shows the TPA results for the SPI-based hydrogels, including hardness, elasticity, chewability, and cohesion. For the Con, SOC, SK, and SKOC hydrogels, there were significant improvements in hardness and chewability (*p* < 0.05). However, the addition of these agents had a minimal impact on the elasticity and cohesion of the hydrogels. The SKOC hydrogel had the highest hardness and chewiness of 545.29 ± 6.84 g and 460.27 ± 5.22, respectively. Wu et al. [45] also found that the incorporation of chitin nanowhiskers into KGM gels enhanced the mechanical properties of the gels. This phenomenon can be attributed to the integration of KGM into the SPI hydrogel, a process that has been shown to enhance protein β-sheets and the formation of hydrogen bonds between molecules. The incorporation of O-ChNCs into the hydrogel results in their collective dispersion, thereby facilitating interaction with both SPIs and KGM. This interaction contributes to the formation of a more compact network within the hydrogels, as evidenced by the results of the SEM analysis. Consequently, this enhanced network improves the physical properties of the hydrogels [46]. This hydrogel exhibited enhanced intermolecular interaction forces, resulting in a more compact gel network. Compared with previous studies [6], the hardness and chewing properties of SKOC gels were significantly improved, which may provide ideas for the development of gel products in the food industry.

**Figure 5:**
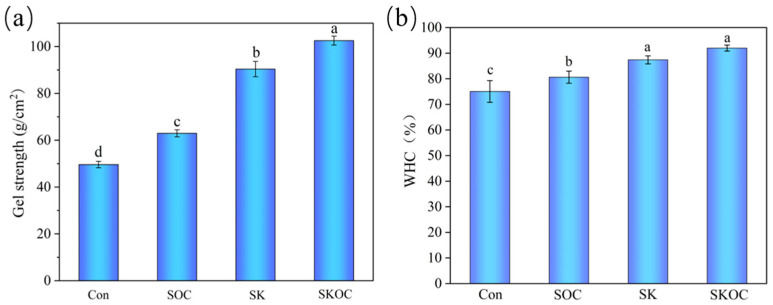
Gel strength (**a**) and WHC (**b**) of Con, SOC, SK, and SKOC hydrogels. Different letters above bars indicate significant differences (*p* < 0.05).

The strength of gels is contingent upon their capacity to withstand external forces, a quality that serves as a crucial metric for evaluating their performance and flavor. The strengths of the SPI-based hydrogels are shown in Figure 5a. The gel strength of hydrogel samples showed significant differences (*p* < 0.05), and the gel strength of the SKOC hydrogel was 102.57 ± 1.91 g/cm^2^. O-ChNCs and KGM could increase the strength of SPI gelation. Additionally, the SKOC hydrogel demonstrated the strongest gel strength, exhibiting high resistance to compression. This was attributed to its unique network structure, which was the most compact among all the samples examined. This finding was consistent with the hardness and chewability test results.

### 3.8. WHC of Hydrogels

A high WHC helps minimize moisture loss, maintain freshness, and provide food with a resilient texture [47]. The WHC of the SPI-based hydrogels is shown in Figure 5b. The SPI-based hydrogels exhibited high WHC (>75%). The WHC of the SOC, SK, and SKOC hydrogels was significantly higher than those of the Con hydrogels (*p* < 0.05), and the WHC of the SK and SKOC hydrogels was significantly higher than those of the SOC hydrogels (*p* < 0.05), with the SKOC hydrogels showing the highest WHC. On the one hand, this phenomenon can be accounted for by the presence of hydroxyl groups in the O-ChNCs and KGM, which facilitate interactions with water molecules, thereby enhancing the WHC of the hydrogel [3,48]. On the other hand, the addition of O-ChNCs and KGM to the hydrogels enhanced the stability of the 3D network structure, leading to a more compact network structure [26]. These observations were consistent with the SEM results, which showed that the addition of O-ChNCs and KGM resulted in a more compact microstructure and smaller pore size of the composite hydrogels.

### 3.9. Molecular Interactions of Hydrogels

**Figure 6:**
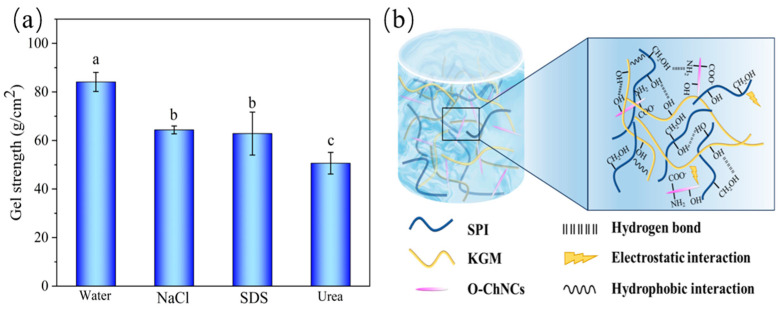
Gel strength of SKOC hydrogels after soaking with distilled water, NaCl solution, urea solution, and SDS solution (**a**), different letters above bars indicate significant differences (*p* < 0.05). Schematic illustrations of potential mechanism of composite hydrogel formation (**b**).

The gel strengths of the SKOC hydrogels after soaking in distilled water, NaCl, urea, and SDS solutions are shown in Figure 6a. The gel strengths of the SKOC hydrogels immersed in NaCl, urea, and SDS solutions were significantly weakened as compared to those immersed in water (*p* < 0.05), with the urea solution (50.63 ± 4.66 g/cm^2^) causing the greatest reduction in gel strength. NaCl neutralizes many charged functional groups, weakening the electrostatic interactions [49]. The hydrophobic terminus of SDS links the hydrophobic groups on the molecule, thereby weakening the hydrophobic interactions. Urea can disrupt hydrogen bonding interactions within or between macromolecules. Hydrogen bonding may be dominant in the network structure of SKOC hydrogels, which is consistent with the FTIR results.

The possible formation mechanisms of the SKOC hydrogels are shown in Figure 6b. The incorporation of KGM into SPI hydrogels improved protein β-folding. The hydroxyl groups on the surface of KGM and the amino and carboxyl groups of SPIs were subjected to intermolecular physical interactions (hydrogen bonding, electrostatic interactions, and hydrophobic interactions). Meanwhile, O-ChNCs has good dispersibility and abundant hydroxyl groups, which can be uniformly distributed in the gel matrix and form interfacial forces with the gel matrix. Therefore, the viscoelasticity, WHC, and gel strength of the composite hydrogel were improved.

## 4. Conclusions

In this study, the incorporation of O-ChNCs and KGM was investigated to enhance the structure and properties of SPI-based hydrogels. The incorporation of KGM into large SPI hydrogels promoted the transformation of the protein’s secondary structure, while KGM promoted the formation of intermolecular interactions with protein molecules to a certain extent. O-ChNCs promoted the entanglement of chains through the filling effect, the formation of hydrogen bonding with the gel matrix, and electrostatic interactions, all of which further contributed to the construction of dense and homogeneous three-dimensional gel networks, so the viscoelasticity of composite gels and the WHC were improved. The SKOC hydrogel had the best physical properties, and the gel strength and hardness were 2.07 and 2.69 times higher than those of the Con hydrogels, respectively. Further studies can explore the addition of vegetable oil to prepare fat substitutes with good mechanical properties and color by using SKOC hydrogel as a matrix.

## Figures and Tables

**Table 1:** Composition of hydrogel samples.

Samples (*w*/*v*)	SPI (g)	KGM (g)	O-ChNCs (g)
Con	16.00	0.00	0.00
SOC	16.00	0.00	0.23
SK	14.50	1.50	0.00
SKOC	14.50	1.50	0.23

**Table 2:** Color of Con, SOC, SK, and SKOC hydrogels.

Samples	L*	a*	b*	Whiteness
Con	31.29 ± 0.32 ^a^	−0.91 ± 0.03 ^a^	11.39 ± 0.64 ^a^	30.45 ± 0.28 ^a^
SOC	30.75 ± 0.14 ^a^	−1.27 ± 0.19 ^a^	10.44 ± 0.07 ^b^	29.97 ± 0.11 ^b^
SK	27.21 ± 0.53 ^b^	−1.28 ± 0.34 ^a^	9.25 ± 0.23 ^c^	26.79 ± 0.44 ^c^
SKOC	26.99 ± 0.18 ^b^	−1.19 ± 0.06 ^a^	8.57 ± 0.27 ^c^	26.54 ± 0.14 ^c^

Mean ± SD values in the same column with different superscript letters are significantly different (*p* < 0.05).

**Table 3:** Mechanical properties of Con, SOC, SK, and SKOC hydrogels.

Samples	Hardness (g)	Springiness	Gumminess	Chewiness
Con	202.53 ± 8.41 ^d^	0.94 ± 0.01 ^b^	0.90 ± 0.01 ^a^	168.82 ± 0.91 ^c^
SOC	236.18 ± 2.97 ^c^	0.95 ± 0.01 ^ab^	0.86 ± 0.03 ^a^	191.22 ± 8.45 ^c^
SK	495.15 ± 14.42 ^b^	0.95 ± 0.01 ^ab^	0.90 ± 0.02 ^a^	439.63 ± 6.88 ^b^
SKOC	545.29 ± 6.84 ^a^	0.96 ± 0.01 ^a^	0.86 ± 0.03 ^a^	460.27 ± 5.22 ^a^

Mean ± SD values in the same column with different superscript letters are significantly different (*p* < 0.05).

## Data Availability

The raw data supporting the conclusions of this article will be made available by the authors on request.
